# Persistent High Long-term Excess Mortality After Elective AAA Repair Especially in Women: A Large Population-based Study

**DOI:** 10.1097/SLA.0000000000006044

**Published:** 2023-07-27

**Authors:** Ruth M.A. Bulder, Joost R. van der Vorst, Jan van Schaik, Ajda Bedene, Willem M. Lijfering, Esther Bastiaannet, Jaap F. Hamming, Jan H.N. Lindeman

**Affiliations:** * Department of Vascular Surgery, Leiden University Medical Center, Leiden, The Netherlands; † Department of Clinical Epidemiology, Leiden University Medical Center, Leiden, The Netherlands; ‡ Epidemiology, Biostatistics and Prevention Institute, University of Zurich, Zurich, Switzerland

**Keywords:** AAA, abdominal aortic aneurysm, age differences, cardiovascular risk management, competing risk of death analysis, CVRM, relative survival, sex differences, time trends, women

## Abstract

**Objective:**

The aim of this time-trend analysis is to estimate long-term excess mortality and associated cardiovascular risk for abdominal aortic aneurysm (AAA) patients after elective repair while addressing the changes in AAA management and patient selection over time.

**Background:**

Despite the intensification of endovascular aneurysm repair and cardiovascular risk management, Swedish population data suggest that AAA patients retain a persistently high long-term mortality after elective repair. The question is whether this reflects suboptimal treatment, a changing patient population over time, or a national phenomenon.

**Methods:**

Nationwide time-trend analysis including 40,730 patients (87% men) following elective AAA repair between 1995 and 2017. Three timeframes were compared, each reflecting changes in the use of endovascular aneurysm repair and intensification of cardiovascular risk management. Relative survival analyses were used to estimate disease-specific excess mortality. Competing risk of death analysis evaluated the risk of cardiovascular versus noncardiovascular death. Sensitivity analysis evaluated the impact of changes in patient selection over time.

**Results:**

Short-term excess mortality significantly improved over time. Long-term excess mortality remained high with a doubled mortality risk for women (relative excess risk=1.87, 95% CI: 1.73–2.02). Excess mortality did not differ between age categories. The risk of cardiovascular versus noncardiovascular death remained similar over time, with a higher risk of cardiovascular death for women. Changes in patient population (ie, older and more comorbid patients in the latter period) marginally impacted excess mortality (2%).

**Conclusions:**

Despite changes in AAA care, patients retain a high long-term excess mortality after elective repair with a persistent high cardiovascular mortality risk. In this, a clear sex – but no age – disparity stands out.

A meta-analysis of long-term survival following elective abdominal aortic aneurysm (AAA) repair reports a persistently high excess mortality after repair.^1^ Swedish population data imply a profound sex difference and suggest that excess mortality has not improved over the last 2 decades. A notable finding considering the advances in surgical care such as the intensification of endovascular aneurysm repair (EVAR) and cardiovascular risk management (CVRM).^2^

Although the persistently compromised life expectancy following elective AAA repair may imply that AAA patients are still suboptimally treated for their long-term mortality risk. It cannot be excluded that it reflects epidemiological changes in AAA care with more comorbid and/or older patients receiving repair in the current endovascular era.^3^ Or that AAA disease may be associated with a residual mortality that is not fully amendable to CVRM. Finally, it cannot be excluded that the apparent lack of survival improvement observed in the Swedish population reflects a national phenomenon. The more so because a recent analysis of Danish population data implied improved survival and cardiovascular (CV) outcomes for AAA patients over time.^4,5^

The aim of this time-trend analysis is to evaluate long-term excess mortality and its associated CV risk for AAA patients after elective repair, while addressing the changes in AAA management (ie, the increase in EVAR and CVRM) and epidemiological shifts (ie, older and more comorbid patients) over time. Therefore, a relative survival analysis was used to estimate disease-specific excess mortality, as well as a competing risk of death analysis to evaluate the risk of CV versus non-CV death over time.

## METHODS

### Study Population

Included patients underwent primary elective AAA repair in The Netherlands between 1995 and 2017.^6^ Ruptured AAAs were excluded (Supplemental Digital Content 1, Table 1, http://links.lww.com/SLA/E782). Comorbidity burden was estimated through the Charlson Comorbidity Index (CCI) and included all diagnoses registered at the same moment as AAA repair.^7^ Prescriptions for CVRM were extracted 1 year before repair to ensure preventive risk management (Supplemental Digital Content 1, Table 1, http://links.lww.com/SLA/E782). The date and cause of death were extracted until December 31, 2018 (Supplemental Digital Content 1, Table 1, http://links.lww.com/SLA/E782).^8^ Three predefined periods were created, each reflecting clear contrasts in procedural an CVRM changes. In period 1 (1995–2000) open repair and rudimentary CVRM dominated; period 2 (2001–2011) was a transition period; and period 3 (2013–2017) saw an EVAR-first strategy and maximal implementation of CVRM.^9–11^ A more detailed prescription of the methods and period choice is provided in the Supplemental Material (Supplemental Digital Content 1, http://links.lww.com/SLA/E782).

### Relative Survival and Competing Risk of Death Analysis

Relative survival, rather than crude survival, was applied to (1) evaluate disease-specific mortality, and (2) to adjust for sex-related, age-related, and time-related differences in life expectancy.^12,13^ A competing risk of death analysis was performed to estimate possible shifts in the risk of CV versus non-CV death over time.^14^ Sensitivity analyses were performed to estimate the impact of changes in patient age and comorbidity burden on study conclusions. Further details are provided in the Supplemental Material (Supplemental Digital Content 1, http://links.lww.com/SLA/E782).

### Statistical Analysis

All analyses were performed in SPSS, version 26 (IBM) and Stata/SE, version 12.0 (StataCorp). Comparisons between groups were analyzed using standard statistical methods. Relative survival reflects the ratio of the observed survival of the study population (ie, electively treated AAA patients), and the expected survival of the matched (sex, age, and year of operation) general Dutch population.^12,13^ A relative survival below 100% means that the disease-specific survival is lower than that of the reference population (ie, excess mortality). Competing risk of death analyses were performed by estimating the cumulative incidence of CV death versus non-CV death (subdistribution hazard ratios, tested by Fine and Gray models).^14^ A detailed description of the statistical analysis is provided in the Supplemental Material (Supplemental Digital Content 1, http://links.lww.com/SLA/E782).

## RESULTS

### Time Trends in Patient Demographics

Between 1995 and 2017, 40,730 patients (86.6% men) underwent elective AAA repair. Clear demographic changes occurred over time (Table 1), including an increase in the proportion of women (from 12.1% to 15.2%), and a ∼2-year increase in mean age at repair (*P*<0.001). Moreover, the CCI increased for men in all age categories, and for women in older age categories (70–74 and >80 years).

**TABLE 1:** Demographics of Patients Treated With Elective AAA Repair Per Period by Sex and Age

	Period 1 (1995–2000)	Period 2 (2001–2011)	Period 3 (2012–2017)	*P*
Elective repair, n (%)	11,096	18,731	10,903	NA
Men	9749 (87.86)	16261 (86.81)	9247 (84.81)	
<65	2280 (20.55)	3117 (16.64)	1398 (12.82)	
65–70	2212 (19.94)	3165 (16.90)	1826 (16.75)	
71–74	2619 (23.60)	4113 (21.96)	2207 (20.24)	
75–79	1846 (16.64)	3702 (19.76)	2054 (18.84)	
>80	792 (7.14)	2164 (11.55)	1762 (16.16)	
Women	1347 (12.14)	2470 (13.19)	1656 (15.19)	
<65	230 (2.07)	331 (1.77)	197 (1.81)	
65–70	240 (2.16)	388 (2.07)	280 (2.57)	
71–74	358 (3.23)	641 (3.42)	362 (3.32)	
75–79	332 (2.99)	659 (3.52)	431 (3.95)	
>80	187 (1.69)	451(2.41)	386 (3.54)	
Age at time repair [mean (SD)]	70.0 (7.5)	71.4 (7.7)	72.3 (7.7)	0.000
Men	69.7 (7.4)	71.2 (7.6)	72.4 (7.7)	0.000
<65	59.7 (4.2)	59.7 (4.1)	60.0 (3.8)	0.004
65–70	67.1 (1.4)	67.2 (1.4)	67.2 (1.4)	0.263
71–74	72.0 (1.4)	72.0 (1.4)	71.9 (1.4)	0.126
75–79	76.7 (1.4)	76.8 (1.4)	77.0 (1.4)	0.000
>80	82.4 (2.3)	82.5 (2.4)	82.9 (2.6)	0.000
Women	71.7 (8.0)	72.8 (7.9)	73.6 (8.0)	0.000
< 65	58.9 (5.4)	58.9 (5.9)	59.1 (6.2)	0.944
65–70	67.2 (1.3)	67.1 (1.4)	67.3 (1.4)	0.079
71–74	72.1 (1.4)	72.1 (1.4)	72.1 (1.4)	0.987
75–79	76.9 (1.4)	76.9 (1.4)	76.9 (1.4)	0.730
>80	83.0 (2.7)	82.9 (2.8)	83.1 (2.7)	0.369
CCI score				
Men, n (%)				
<65				0.001
0	1274 (55.9)	1687 (54.1)	821 (58.7)	
1	720 (31.6)	1061 (34.0)	379 (27.1)	
2	192 (8.4)	240 (7.7)	128 (9.2)	
>3	94 (4.1)	129 (4.1)	70 (5.0)	
65–70				0.000
0	1400 (51.3)	2088 (52.5)	1237 (54.0)	
1	923 (33.8)	1319 (33.2)	651 (28.4)	
2	252 (9.2)	331 (8.3)	248 (10.8)	
>3	154 (5.7)	238 (6.0)	157 (6.8)	
71–74				0.000
0	1037 (49.3)	1672 (50.6)	899 (51.7)	
1	698 (33.2)	1076 (32.6)	465 (26.7)	
2	227 (10.8)	306 (9.3)	220 (12.6)	
>3	140 (6.7)	248 (7.5)	156 (9.0)	
75–79				0.001
0	903 (48.9)	1888 (51.0)	1045 (50.9)	
1	597 (32.3)	1153 (31.2)	566 (27.6)	
2	208 (11.3)	365 (9.8)	260 (12.7)	
>3	138 (7.5)	296 (8.0)	183 (8.9)	
>80				0.000
0	352 (44.4)	1128 (52.1)	905 (51.4)	
1	280 (35.4)	664 (30.7)	464 (26.3)	
2	97 (12.2)	187 (8.6)	226 (12.8)	
>3	63 (8.0)	185 (8.6)	167 (9.5)	
Women				
<65				0.379
0	124 (53.9)	161 (48.6)	101 (51.3)	
1	78 (33.9)	130 (39.3)	62 (31.5)	
2	19 (8.3)	24 (7.3)	22 (11.2)	
>3	9 (3.9)	16 (4.8)	12 (6.1)	
65–70				0.142
0	153 (51.2)	239 (48.0)	169 (48.3)	
1	93 (31.1)	174 (35.0)	100 (28.6)	
2	30 (10.0)	56 (11.2)	45 (12.8)	
>3	23 (7.7)	29 (5.8)	36 (10.3)	
71–74				0.024
0	143 (47.8)	241 (45.4)	126 (43.2)	
1	111 (37.1)	199 (37.5)	90 (30.8)	
2	26 (8.7)	53 (10.0)	42 (14.4)	
>3	19 (6.4)	38 (7.2)	34 (11.6)	
75–79				0.077
0	153 (46.1)	316 (48.0)	210 (48.7)	
1	119 (35.8)	228 (34.6)	133 (30.9)	
2	42 (12.7)	65 (9.8)	41 (9.5)	
>3	18 (5.4)	50 (7.6)	47 (10.9)	
>80				0.016
0	96 (51.3)	217 (48.1)	212 (54.9)	
1	58 (31.0)	154 (34.2)	86 (22.3)	
2	18 (9.6)	45 (10.0)	52 (13.5)	
>3	15 (8.0)	35 (7.7)	36 (9.3)	

Data are presented as number (%) unless indicated otherwise.

NA indicates not available.

### Time Trends in CVRM

**FIGURE 1:**
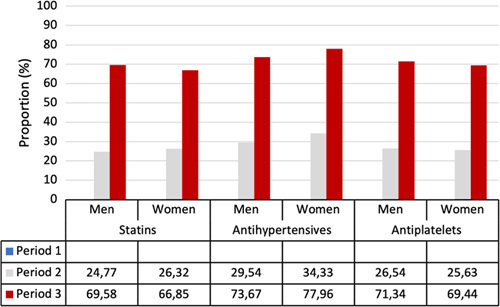
Time trends of the implementation of pharmaceutical CVRM, stratified by sex.

Detailed information regarding medical CVRM was available for the years 2006 to 2017 and summarized in Figure 1. Over time, the proportion of patients with at least 1 CVRM prescription increased: statins from 24.8% to 69.6% and 26.3% to 66.9%, antihypertensive from 29.5% to 73.7% and 34.3% to 78.0%, and antiplatelets from 26.5% to 71.3% and 25.6% to 69.4%, for men and women, respectively.

### Time Trends in Excess Mortality (Relative Survival)

**FIGURE 2:**
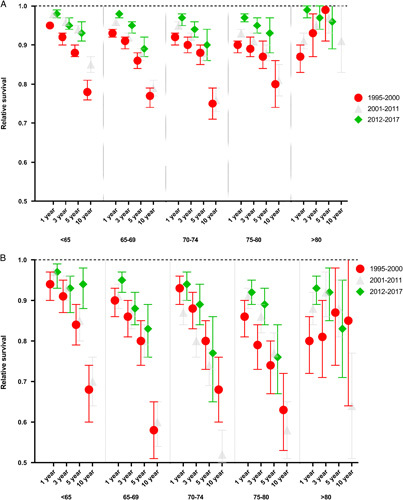
Relative survival (1, 3, 5, 10 years) for men (A) and women (B) stratified by age. Note that 10-year relative survival could only be calculated for periods 1 and 2 due to the restricted follow-up in period 3.

Short-term (1 year) relative survival improved for men, but not for women, over time. Long-term relative survival (3, 5, and 10 years) remained stably impaired over time, with a clear survival disadvantage for women (Fig. 2, Supplemental Digital Content 1, Table 2, http://links.lww.com/SLA/E782). In fact, compared with men, women had a doubled mortality risk after correction for age and CCI scores (relative excess risk=1.87, 95% CI: 1.73–2.02). There were no differences in relative survival between age categories.

### Time Trends in Competing Risk Analysis of CV Versus Non-CV Death

Cause of death analysis implies a higher risk for CV death in the electively repaired AAA population compared with the general population (Supplemental Digital Content 1, Figure 1, http://links.lww.com/SLA/E782).

**FIGURE 3:**
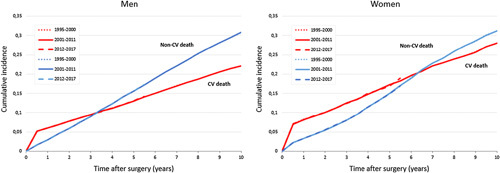
Cumulative incidence of CV versus non-CV mortality over time stratified by sex.

A competing risk analysis was performed to estimate the risk of CV versus non-CV mortality over time. This showed no change in cumulative incidence of CV versus non-CV death for all age categories and for both sexes over time. Throughout the first years after repair, the risk of CV death exceeded that of non-CV death. The dominance of CV death persisted for a longer time after surgery in women (to 6.5 years) than in men (to 3.5 years) (Fig. 3, Supplemental Digital Content 1, Table 3, http://links.lww.com/SLA/E782, Supplemental Digital Content 1, Figure 2, http://links.lww.com/SLA/E782).

### The Impact of Changes in Patient Characteristics on Study Outcomes

From periods 1 to 3, the mean age at the time of repair increased to 2.7 years for men and 1.9 years for women (both *P*<0.001). Comorbidity scores increased for men in all age categories and for women in age categories 70 to 74 and >80 years. To estimate the impact of the lower repair threshold, resulting in an increase in the proportion of older and/or patients with greater comorbidity in the more recent time frame, stratified analyses by age, and sensitivity analyses addressing different degrees of comorbidity, were undertaken. Outcome comparison of age categories showed similar survival rates and equal risks of CV death (Fig. 2, Supplemental Digital Content 1, Figure 2, http://links.lww.com/SLA/E782). Sensitivity analyses addressing CCI showed a higher relative excess risk of death for patients with a higher CCI score, and a more pronounced impact of comorbidities on survival in older patients (Supplemental Digital Content 1, Table 4, http://links.lww.com/SLA/E782). Censoring all patients with a CCI score ≥3 receiving repair in period 3, implied a modest impact of changes in patient comorbidity on the relative survival (∼2%) for men (<65, 65–69, 75–80 years) and for younger women (<65 years) (Table 2).

**TABLE 2:** Sensitivity Analysis

	Period 1 (1995–2000)	Period 3 (2012–2017)	
	Entire cohort	Entire cohort	Exclusion CCI >3
Men			
<65	0.88 (0.87–0.90)	0.93 (0.91–0.96)	0.94 (0.91–0.96)
65–70	0.86 (0.84–0.88)	0.89 (0.87–0.92)	0.92 (0.89–0.95)
71–74	0.88 (0.85–0.90)	0.90 (0.86–0.94)	0.93 (0.88–0.96)
75–79	0.87 (0.84–0.91)	0.93 (0.88–0.97)	0.95 (0.91–0.99)
>80	0.99 (0.91–1.06)	0.96 (0.89–1.02)	1.00 (0.92–1.07)
Women			
<65	0.84 (0.79–0.89)	0.94 (0.88–0.98)	0.95 (0.90–0.99)
65–70	0.80 (0.74–0.85)	0.83 (0.76–0.89)	0.86 (0.78–0.92)
71–74	0.80 (0.73–0.85)	0.77 (0.65–0.86)	0.79 (0.67–0.88)
75–79	0.74 (0.67–0.80)	0.76 (0.67–0.84)	0.79 (0.70–0.87)
>80	0.85 (0.64–1.09)	0.83 (0.71–0.95)	0.88 (0.75–1.00)

Data represent relative survival with 95% CI.

Five-year relative survival of entire patient cohort versus patient cohort with exclusion of patients with CCI>3.

## DISCUSSION

In this 25-year nationwide study, short-term mortality after elective AAA repair was improved, as expected. Long-term mortality was stable, despite an aging patient population with increasing comorbidity. Long-term excess mortality, compared with a sex-matched and age-matched general population, however, remained persistently high. The risk of CV death did not decrease over time despite the intensification of pharmaceutical CVRM. Profound sex but no age disparity stands out with a doubled excess mortality risk for women compared with men.

Despite improvements in short-term survival over time due to improvements in surgical outcomes, long-term survival remained persistently impaired. To put this in perspective, the excess mortality of elective AAA patients equals that or is higher than, reported for most malignancies.^15^ The absence of long-term survival benefit could be explained the gradual lowering of the threshold for repair, which resulted in older and more comorbid patients considered eligible for repair in the current era (chronological bias). In this study, this is reflected by an increase in CCI, a doubling of the proportion of octogenarians, and a two-year increase in mean age at repair over the study period. The potential impact of these changes in a patient selection over time was addressed by age-stratified and sensitivity analyses. This showed that increased age did not correspond with lower survival. Associations were found between higher CCI scores and a higher risk of excess mortality (Supplemental Digital Content 1, Table 4, http://links.lww.com/SLA/E782). To evaluate the impact of higher CCI scores on the excess long-term mortality, a sensitivity analysis censoring all with a CCI ≥3 in period 3 was performed. This showed a marginal increase in relative survival of 2% (predominately men), yet relative survival remained severely impaired (Table 2). Therefore, the persistently high excess mortality only marginally relates to an increase in comorbidity burden. The observation that there were no differences in excess mortality between age categories implies that the existence of an AAA reflects overall vulnerability, resulting in a univocally high mortality risk, regardless of whether a patient is 65 or 85 years old.

To gain a better understanding of possibly underlying causes for the excess long-term mortality, overall causes of death were evaluated. This implied a high CV cause of death (without AAA rupture) in the electively repaired AAA population (∼50%) compared with the general population. A result confirmed by other studies.^16^ To evaluate to what extent the intensification of CVRM over time influences the risk of CV mortality, a competing risk of death analysis was performed. Hypothetically, intensification of CVRM should decrease the risk for CV death, thereby exposing the patient to other competing mortality risks (eg, malignancy), and thus potentially masking a beneficial effect of CVRM on overall survival. This analysis showed an unchanged CV mortality risk over time, as well as a particularly high CV risk for AAA patients in the first years after surgery. Therefore, it is suggested that the broader implementation of CVRM has a limited impact on CV mortality risk (on population level).

The apparently limited impact of CVRM on CV mortality risk may reflect relative undertreatment of CV risk in electively repaired AAA patients. Despite consensus endorsing maximum CVRM in those patients, studies show that half of the AAA patients still do not receive optimal CVRM.^17,18^ Moreover, AAA patients could be relatively resistant to traditional CVRM, as risk factors for AAA disease are different from traditional (atherosclerotic) CV risk factors.^19^ The unchanged excess mortality could reflect the dominant role of smoking in AAA patients compared with the general population (Supplemental Digital Content 1, Table 5, http://links.lww.com/SLA/E782). Yet, prior research showed that the impact of smoking is minimal on disease-specific excess mortality for diseases highly associated with excessive smoking.^20^ Alternatively, although the effectiveness of CVRM on nonfatal CV events has been firmly established, an effect on survival is unclear, as literature shows heterogenous results on the effect of CV medication of life expectancy.^21,22^

This study showed a worrisome persistently high excess mortality for women. While inferior survival outcomes for women have been outlined previously, the basis for this sex disparity remains unclear.^23–25^ This study indicates that the disparity is not explained by a higher age of women at the time of repair, as there were no significant survival differences between age categories. Alternative explanations include a higher allostatic load or comorbidity profile of women.^26^ However, the results of this study portray equal CCI scores for men and women, with an even lower effect size of CCI scores on survival in women (Supplemental Digital Content 1, Table 4, http://links.lww.com/SLA/E782). This is supported by a meta-analysis reporting fewer baseline comorbidities for women.^27^ The high CV mortality of women, which persists for a longer time after surgery, points to the involvement of CV risk factors in the excess mortality for women. In this context, poorer profiles of female AAA patients are worrisome and suggest that women receive suboptimal treatment for their CV risk. This appears a general phenomenon because women, even when correctly diagnosed, are often undertreated for their CV risk factors.^28^ Last, in contrast to men, the absence of a decline in tobacco use in women could explain the sex-dependent survival differences.^29^

Our data for the Dutch population confirm and extend Swedish population data, which showed a persistent impaired survival of AAA patients who underwent elective repair (Supplemental Digital Content 1, Figure 3, http://links.lww.com/SLA/E782).^2^ This conclusion contrast with a recent Danish study, reporting a decrease in overall mortality and CV risk in patients diagnosed with AAA over time.^4^ There are several explanations for this apparent discrepancy. First, the Danish study describes a heterogenous incomparable patient population that included both patients with either intact AAA or ruptured AAA, without mentioning surgery. Moreover, the follow-up in the Danish study was limited to 2 years, and therefore the improved survival of the Danish study population presumably reflects the improvements in short-term survival observed in the current study. Given the comparable excess mortality rates observed in both the Dutch and Swedish cohorts, it is unlikely that the persistently high excess mortality reflects a national phenomenon.

### Strengths and Limitations

The first strength of this study is that is relies on a large nationwide registry, with a high reported validity (84%–99%), and covers an extensive period of 25 years. A second strength is the fact that both the introduction of EVAR and CVRM occurred in well-defined timeframes. This facilitated our time-trend analysis. Availability of data from 1995 to 2017 allowed for comparison of a period with almost no EVAR and CVRM, with a period of EVAR-first strategy and extensive CVRM. Application of relative survival analyses allowed to estimate disease-specific excess mortality while accounting for the changes in patient characteristics over time (ie, more female and older patients). As a result of an aging population and lower intervention threshold, a growing number of elderly patients is, and will be, considered eligible for AAA repair.^3^ Age differences exert important effects on survival comparisons but are often not considered in (traditional) survival analysis. Older patients have a higher a priori risk of death. Therefore, comparing overall crude survival differences between younger and older patients introduces bias (immortal time bias).^30^ Relative survival analysis, which corrects for age differences in (a priori) life expectancy, overcomes this. Furthermore, to the best of our knowledge, this is the first study to apply a competing risk analysis for CV mortality in AAA patients. As opposed to traditional survival analysis, competing risk analysis addresses the competitive nature of multiple causes to the same event.

This study has some limitations inherent to the retrospective design and the use of registry data such as the quality of coding.^31,32^ The use of general procedural codes prohibited a distinction between EVAR and open repair. However, long-term survival rates are reported to be similar for EVAR and open repair and were not the focus of this study.^2,3^ Cause of death registration is prone to bias and misclassification, hence the interpretation of specific causes of death requires caution. Analyses of main categories of causes of death are less prone to misclassification. This study aimed to address time-dependent differences and it is unlikely that there are differences/bias in the cause of death registration over time. Furthermore, stratification of results inevitably leads to a restricted number of patients and therefore larger 95% CIs. This hampers the detection of more subtle differences in women. Last, while we were unable to demonstrate a benefit of CVRM on CV death, a potential benefit of CVRM on nonfatal CV events (prevention or postponement of myocardial infarction or stroke), was considered beyond the scope of this study.

## CONCLUSIONS

Although improvements in surgical care resulted in significantly lower short-term mortality and broadened the spectrum of patients eligible for repair, AAA patients, especially women, retain a high long-term excess mortality risk. Despite the intensification of CVRM, the CV mortality risk remains the primary cause of death. The persistently high excess mortality appears largely independent of changes in patient selection. Future studies should focus on high sex-dependent excess mortality and strategies to reduce accompanied comorbidity risks.

## Supplementary Material

SUPPLEMENTARY MATERIAL

## DISCUSSANT

### Slawomir Nazarewski (Warsaw, Poland)

lnfrarenal abdominal aortic aneurysms (AAAs) are the most common true aneurysms in around 1% of the general population, aged 55 and older. The prevalence of AAA rises by 2-4% every decade after that. The vast majority of AAAs (up to 75%) are asymptomatic and are frequently discovered inadvertently on abdominal imaging for another reason. Elective AAA repair is performed to prevent aneurysm rupture, which has an 80% fatality rate and is the tenth highest cause of death in men aged 65 to 74 years in the United States. Well-defined clinical risk factors are associated with the pathogenesis of AAAs, including older age, male gender, smoking, Caucasian race, atherosclerosis, and hypertension. A decreased risk of AAA is associated with the female gender, diabetes, and non-Caucasian race.

Aneurysm repair can be accomplished using open surgical or endovascular (EVAR) techniques. The choice between these methods should be personalized, taking into account the patient’s age, risk factors for perioperative morbidity and mortality, anatomic factors, and finally, the experience of the surgeon. EVAR is associated with a lower risk of perioperative morbidity, compared to open repair for asymptomatic, symptomatic, and ruptured AAA. Long-term mortality following elective AAA repair is not significantly different between the techniques.

The authors of the manuscript have analyzed a very large population based group of patients (over 40K) operated electively in the Netherlands on AAA over a period of over 25 years. The analysis was stratified into three time periods. The aim of this time-trend analysis was to estimate long-term excess mortality and associated cardiovascular risk for AAA patients after elective repair, while addressing the changes in AAA management, base medical treatment (e.g. intensification of cardiovascular (CV) risk management), and patient selection over time.

The authors stated that short-term excess-mortality significantly improved over time, i.e. due to the introduction of the minimally invasive EVAR procedure. However, long-term excess-mortality remained high, with a doubled mortality risk for women. Excess mortality did not differ between age categories. They found that the risk of cardiovascular versus non-CV death remained similar over time, with a higher risk of cardiovascular death for women. They also underlined that changes in patient population marginally impacted excess-mortality. The authors concluded that, despite changes in AAA care, patients retained a high long-term excess-mortality after elective repair with a persistent high CV mortality risk. As such, a clear sex, not age, disparity appeared.

As we know, patients with an AAA have an increased burden of atherosclerotic comorbidities and several other CV risk factors. Their risk of CV complications is increased compared to the normal population. Long-term survival after AAA surgery is affected by age at the time of repair, AAA diameter, and sex. A case control analysis from the UK, comparing patients who had AAA repair, revealed a 5-year freedom from an adverse CV event rate of 86%, compared to 93% for controls.

I have the following comments and questions: First, this is a large population-based study, but it is limited to the Dutch population. Is it really a nationwide Dutch phenomenon?

Second, is it worth comparing the results with the general population and the non-ruptured AAA patients who were treated conservatively?

Finally, further prospective trials and studies are needed to explain the phenomenon mentioned above, especially as other data revealed improvement outcomes after AAA repair with no gender-dependency.

### Response From Ruth Bulder (Leiden, The Netherlands)

Thank you for your remarks. Regarding the first question, the data presented today pertains to the Dutch AAA population. However, as mentioned in the discussion, we observed similar results in the Swedish AAA population. Therefore, we believe that the results are not limited to a national phenomenon, but that, unfortunately, it truthfully reflects the AAA population.

To address the second question, comparison to the general population is done with a relative survival analysis, which was used in the current study. Comparison to non-ruptured AAAs treated conservatively is interestingly, as it questions the effectiveness of elective repair, but it is a different population compared to the surgically treated AAAs. Generally, when an aneurysm reaches 55 mm, there is an indication for surgery. When comparing patients with AAAs (>55 mm) who undergo surgery versus those treated conservatively, you introduce confounding by indication because the decision for repair is based on the patient’s co-morbidity profile (i.e., patients who are too frail will be denied surgery). Therefore, retrospective comparison is challenging. There have been four RCTs in the field of elective AAA repair, one of which looked at the decision to either operate with EVAR or treat conservatively. When we calculate the relative survival for this RCT, it is lower than the relative survival rates observed in the Swedish or Dutch population. This underlines the necessity for surgery, but also highlights the need to implement additional measures to further improve the life expectancy of these patients.

Regarding the third question, we acknowledge the need for further research to determine the reasons for the persistently high long-term excess mortality.

### Wolf Bechstein (Frankfurt Am Main, Germany)

Data from the US show that women are at a higher risk of cardiovascular disease for several reasons, including atypical presentation of symptoms and impaired access to diagnostic and interventional cardiology. Women often experience different symptoms of coronary artery disease from what men experience. Women’s symptoms are often more subtle. Do you think that this could be one of the reasons why coronary artery disease is one of the main causes of death in patients with cardiovascular disease in the long-term?

### Response From Ruth Bulder (Leiden, The Netherlands)

Yes, I think that this is true. Over time, more research has been conducted on women. One explanation for the severe sex-deficit in elective AAA repair is that we think that women have a higher (cardiovascular) co-morbidity profile at the time of repair. Interestingly, a recent meta-analysis showed that when you look at women pre-operatively, they are reported to have a lower co-morbidity profile. However, I believe this serves as an example of a potential lack of awareness regarding the comorbidities experienced by women. So, now that we know that women are doing far worse, we need to pay special attention to them, in order to provide them with a better level of treatment

### Frederic Ris (Geneva, Switzerland)

Indeed, we know that women with chest pains are sent to psychiatrists more often than men. Did you look at the data to see whether the women who had a lower survival rate underwent the same treatment as the men?

### Response From Ruth Bulder (Leiden, The Netherlands)

This could theoretically be possible, but we haven’t looked at this.
