# Supplementary material for: Persistent High Long-term Excess Mortality After Elective AAA Repair Especially in Women: A Large Population-based Study
Source: Ann Surg. 2023 Jul 27;278(5):815–22. doi: 10.1097/SLA.0000000000006044 (PMC10549885; doi:10.1097/SLA.0000000000006044)
Supplement: SUPPLEMENTARY MATERIAL [file sla-278-00815-s001.docx]

**Supplemental Material**

**METHODS**

*Data sources*

Data was obtained from national Dutch registries which were accessed through Statistics Netherlands: the Population Register (PR), Hospital Discharge Register (HDR), Medication Register (MR), and Cause of Death Register (CDR).^[[1]](#endnote-1)^ The PR contains baseline data of all residents in the Netherlands. The HDR contains data on hospitalisations, including admission- and discharge dates, diagnosis, and (surgical) procedures. The MR includes data of medication prescribed in hospitals and dispensed from outpatient or community pharmacies or in care homes. The CDR registers date of death, and causes of death based on death certificates.^[[2]](#endnote-2)^

All registers include an individual person- and admission- specific identification number assigned to each resident in the Netherlands, which allows data merging on individual patient level from the different registries.

*Data governance*

All linkages and analyses were performed in a secured data environment of Statistics Netherlands. The data handling was completely anonymized, and in full agreement with privacy legislation in the Netherlands. According to the Dutch law, approval from an independent ethics committee is not required for registry-based studies in which specific individuals cannot be identified. This study was conducted according to the STROBE guidelines for reporting of observational cohort studies in epidemiology.^[[3]](#endnote-3)^

*Patient population*

All patients who underwent elective abdominal aortic aneurysm repair between 1995 and 2017 were identified through the Hospital Discharge Register (HDR). Patients were included with a first registered hospitalization with a primary diagnosis of intact AAA (ICD-9: 441E or ICD10: I174) and subsequent AAA repair (CCV/CBV/ZA codes: **Supplemental Table 1**). Patients with a diagnosis of ruptured AAA (ICD-9: 441D or ICD10: I173) or no procedural code were excluded. Comorbidities included all diagnosis (ICD-9 and ICD-10) registered during the same hospital admission as AAA repair. The comorbidity burden was estimated through the Charlson Comorbidity Index (CCI). The CCI is a weighted score of comorbidities ranging from 0 (no comorbidities) to 6 (extensive comorbidities).^[[4]](#endnote-4)^^[[5]](#endnote-5)^^[[6]](#endnote-6)^ Before 2005 and after 2014 registration in the HDR was mandatory, but voluntary for the 2005-2013 interval. As result, the number of participating hospitals in the HDR transiently declined between 2005-2014. The estimated proportion of missing cases carried from 5 to 25 per cent between 2005-2014. Missing cases are considered missing at random, and are therefore not likely to influence results. In 2012 there was a transition in registrations, as such there is no data available for this year.

Prescriptions for cardiovascular risk management were extracted from the MR based on ATC4 codes (**Supplemental Table 1**). To ensure that prescriptions represented secondary CVRM prescriptions, data was extracted one year prior to the elective repair.

Mortality data was obtained from the CDR until 31 December 2018.

*Time-trends*

Three predefined time periods, each reflecting clear contrasts in procedural and CVRM characteristics were created. A first period (1: 1995-2000), dominated by open repair and rudimentary pharmaceutical CVRM; a second period (2: 2001-2011), reflecting the transition from open repair to EVAR and progressive implementation of CVRM; and a third period (3: 2013-2017), with an EVAR-first strategy and full implementation of CVRM. Up to 1994 no EVARs were performed in the Netherlands. From 1995 to 2017 the proportion of EVAR gradually increased from 1.2% to 80%.^[[7]](#endnote-7)^^[[8]](#endnote-8)^^[[9]](#endnote-9)^ The proportion of CVRM significantly increased from 2006 until 2017 (P = 0.000). The proportion of patients with at least one CVRM prescription increased: statins from 24.8%-69.6% and 26.3%-66.9%, antihypertensive from 29.5%-73.7% and 34.3%-78.0%, and antiplatelets from 26.5%-71.3% and 25.6%-69.4%, for men and women respectively.

*Outcomes: relative survival and competing risks of death*

The primary outcomes included time-trends in 1- 3- 5- and 10- year(s) relative survival rate(s). Relative survival, rather than crude survival, was chosen to 1) evaluate the disease-specific mortality, and 2) to adjust for sex-, age and time-dependent differences in life-expectancy.^[[10]](#endnote-10)^^[[11]](#endnote-11)^

While relative survival provides an estimate of disease-specific survival, and corrects for changes in demographics, it is a relative measure with the absolute impact being determined by the disease-specific *and* population (background) mortality. As a more tangible illustration of the disease-specific mortality remaining after successful AAA repair, **Supplemental Table 2** was included to summarize the actual observed and expected 5-years mortality, as well as the excess mortality rates.

Secondary outcomes included time-trends in cause of death distribution and a competing risk analysis for cardiovascular versus non-cardiovascular death. Causes of death were determined based on ICD-9 and ICD-10 codes (**Supplemental Table 1**). A competing risk-of-death analysis was performed in order to estimate the risk of cardiovascular death (CV) in the presence of non-CV death.^[[12]](#endnote-12)^ This aspect was considered relevant since changes in intensity of CVRM potentially altered the cardiovascular mortality risk. Hypothetically, implementation of CVRM could lead to a decrease of the cardiovascular mortality, thereby exposing the patient to other (competing) mortality risks and masking a potential beneficial effect of CVRM on overall mortality.

*Sensitivity analysis*

To estimate the potential impact of changes in patient frailty on study conclusions, two sensitivity analyses were performed. Firstly, differences between older and younger patients were evaluated. Secondly, a generalized mixed model (GLM) was applied in order to estimate the impact of comorbidities (CCI index) on relative survival.

*Statistical analysis*

All analyses were performed with SPSS, version 26 (IBM, Amsterdam) and Stata/SE, version 12.0 (StataCorp, College Station, TX, USA). Normality was assessed by histograms. Continuous variables were expressed as means (+SD) or medians (+IQR) and compared using Student's t-test or Mann-Whitney test. Categorical data were expressed as proportions and analysed using the chi-square test. A two-sided p-value of <0.05 was considered statistically significant.

Relative survival was calculated as the ratio of observed survival of the study population (i.e. electively treated AAA patients) and the expected survival of the matched (age, sex, and year of operation) general Dutch population.^RS^ Population life-tables were used to estimate the expected survival.^[[13]](#endnote-13)^ A relative survival below 100% means that the disease-specific survival is lower than expected for the reference population. The relative excess risk (RER) of death was estimated by a multivariate generalized linear model (GLM) with a Poisson distribution based on collapsed relative survival data on specific survival end-points.

Competing risk analyses were performed by estimating the cumulative incidence of CV death throughout 10 years follow-up with non-CV death as competing risk. Cumulative incidence as percentages with corresponding 95% CIs were reported for 1, 3, 5, and 10 years. Subdistribution hazard ratios were reported with 95% CIs to allow for statistical testing (Fine and Gray models).

**Supplemental Table 1.** Codes used for data extraction of patient inclusion, cardiovascular prescriptions, and causes of death.

| DIAGNOSIS | |
| --- | --- |
| **ICD-9 codes** | |
| AAA | 441E |
| **ICD-10 codes** | |
| AAA | I174 |
| PROCEDURE | |
| **CVV codes** | |
|  | 883656 883653 539995 539990 539985 539980 539595 539590 539575 539570 538338 538337 538336 538335 538334 538333 538332 538331 538330 31311 31319 31318 31310 3313 539525 31390 538541 31313 883603 538241883606 31380 538090 538540 538090 538540 538541 538244 88368 538242 538040 |
| **CBV codes** | |
|  | 388932A 333556E333556D 333556C 333555B 333555A 333539B 333539BA 333533B 333533A 333532F 333532E 333532D 333532C 333532B 333532A 333530R 333530Q 333530P 333530N 333530M 333530K 333530J 333530H 333530G 333530C 333530A 333154B 333154A 333153Y 333153X 333153R 333153Q 333153M 333153L 388922 385332 380020 333558 333556 333555 333553 333539 333538 333537 333536 333535 333534 333533 333532 190302 080930 033699 033562 033561 033560 033559 033555 033554 033521 033520 |
| **ZA codes** | |
|  | 190302 080930 080822 080821 080823 033562 33561 033560 033559 033558 033557 033556 033555 033554 033521 033520 |
| MEDICATION | |
| **ATC code** |  |
| Statins | C10A C10B |
| Antihypertensive | C02K C02L C02N C03A C03B C03C C03D C03E C03X C07A C07B C07C C07D C07E C07F C08C C08D C08E C08G C09A C09B C09C C09D C09X C04A |
| Antiplatelets | B01A |
| CAUSES OF DEATH | |
| **ICD-9 codes** | |
| Cardiovascular | 39, 40, 41, 42, 43, 44, 45 |
| Respiratory | 46, 47, 48, 49, 50, 51 |
| Digestive | 52, 53, 54, 55, 56, 57 |
| Malign neoplasm | 14, 15, 16, 17, 19, 20 |
| Renal | 58, 59, 60, 61, 62 |
| Nervous system | 32, 33, 34, 35, 36, 37, 38 |
| Other | All other |
| **ICD-10 codes** | |
| Cardiovascular | I |
| Respiratory | J |
| Digestive | K |
| Malign neoplasm | M |
| Renal | N |
| Nervous system | G |
| Other | All other |

**Supplemental Table 2.** Excess mortality of AAA patients undergoing elective repair between 1995 – 2017, stratified by men (A) and women (B) and age categories.

**A**

|  |  | **< 65** |  |  | **65 - 69** |  |  | **70 - 74** |  |  | **75 - 79** |  |  | **> 80** |  |  |
| --- | --- | --- | --- | --- | --- | --- | --- | --- | --- | --- | --- | --- | --- | --- | --- | --- |
| **Men** |  | Period 1 | Period 2 | Period 3 | Period 1 | Period 2 | Period 3 | Period 1 | Period 2 | Period 3 | Period 1 | Period 2 | Period 3 | Period 1 | Period 2 | Period 3 |
| 1 year | N | 2280 | 3117 | 1398 | 2729 | 3976 | 2293 | 2102 | 3302 | 1740 | 1846 | 3702 | 2054 | 792 | 2164 | 1762 |
|  | Observed mortality | 141 | 100 | 44 | 261 | 254 | 96 | 254 | 276 | 108 | 302 | 434 | 156 | 185 | 394 | 192 |
|  | Expected mortality | 26,2 | 28,7 | 12 | 68,2 | 78,4 | 39,7 | 85,5 | 106,9 | 49,5 | 111,1 | 187,6 | 97,2 | 78,6 | 196,5 | 166,3 |
|  | Excess mortality (O-E) | 114,8 | 71,3 | 32 | 192,8 | 175,6 | 56,3 | 168,5 | 169,1 | 58,5 | 190,9 | 246,4 | 58,8 | 106,4 | 197,5 | 25,7 |
|  | Excess mortality % | 5,04 | 2,29 | 2,29 | 7,06 | 4,42 | 2,46 | 8,02 | 5,12 | 3,36 | 10,34 | 6,66 | 2,86 | 13,43 | 9,13 | 1,46 |
|  | Excess mortality rate | 5,38 | 3,48 | 3,67 | 3,83 | 3,24 | 2,42 | 2,97 | 2,58 | 2,18 | 2,72 | 2,31 | 1,60 | 2,35 | 2,01 | 1,15 |
| 3 year | N | 2073 | 2959 | 1060 | 2364 | 3579 | 1674 | 1743 | 2863 | 1175 | 1421 | 3057 | 1386 | 547 | 1641 | 1081 |
|  | Observed mortality | 62 | 66 | 15 | 93 | 155 | 51 | 107 | 158 | 50 | 101 | 239 | 85 | 55 | 175 | 129 |
|  | Expected mortality | 28,9 | 32,2 | 9,5 | 73,2 | 83,9 | 30,4 | 88,5 | 113 | 37,3 | 111,7 | 194,3 | 72,2 | 75,3 | 191,4 | 106,5 |
|  | Excess mortality (O-E) | 33,1 | 33,8 | 5,5 | 19,8 | 71,1 | 20,6 | 18,5 | 45 | 12,7 | -10,7 | 44,7 | 12,8 | -20,3 | -16,4 | 22,5 |
|  | Excess mortality % | 1,60 | 1,14 | 0,52 | 0,84 | 1,99 | 1,23 | 1,06 | 1,57 | 1,08 | -0,75 | 1,46 | 0,92 | -3,71 | -1,00 | 2,08 |
|  | Excess mortality rate | 2,15 | 2,05 | 1,58 | 1,27 | 1,85 | 1,68 | 1,21 | 1,40 | 1,34 | 0,90 | 1,23 | 1,18 | 0,73 | 0,91 | 1,21 |
| 5 year | N | 1949 | 2823 | 447 | 2150 | 3253 | 674 | 1512 | 2551 | 465 | 1199 | 2566 | 495 | 434 | 1305 | 341 |
|  | Observed mortality | 71 | 56 | 8 | 140 | 161 | 26 | 97 | 198 | 26 | 127 | 229 | 26 | 58 | 179 | 39 |
|  | Expected mortality | 31,5 | 36 | 4,1 | 76,9 | 90,6 | 12,2 | 90,3 | 119,1 | 15,1 | 109,2 | 195,3 | 26,6 | 69,7 | 178,8 | 33,2 |
|  | Excess mortality (O-E) | 39,5 | 20 | 3,9 | 63,1 | 70,4 | 13,8 | 6,7 | 78,9 | 10,9 | 17,8 | 33,7 | -0,6 | -11,7 | 0,2 | 5,8 |
|  | Excess mortality % | 2,03 | 0,71 | 0,87 | 2,93 | 2,16 | 2,05 | 0,44 | 3,09 | 2,34 | 1,48 | 1,31 | -0,12 | -2,70 | 0,02 | 1,70 |
|  | Excess mortality rate | 2,25 | 1,56 | 1,95 | 1,82 | 1,78 | 2,13 | 1,07 | 1,66 | 1,72 | 1,16 | 1,17 | 0,98 | 0,83 | 1,00 | 1,17 |
| 10 year | N | 1587 | 2031 | - | 1524 | 2051 | - | 921 | 1393 | - | 623 | 1200 | - | 191 | 394 | - |
|  | Observed mortality | 79 | 69 | - | 106 | 132 | - | 99 | 134 | - | 90 | 154 | - | 32 | 68 | - |
|  | Expected mortality | 34,9 | 38,7 | - | 77 | 91,9 | - | 78,6 | 106,6 | - | 82,3 | 139 | - | 44,2 | 78,2 | - |
|  | Excess mortality (O-E) | 44,1 | 30,3 | - | 29 | 40,1 | - | 20,4 | 27,4 | - | 7,7 | 15 | - | -12,2 | -10,2 | - |
|  | Excess mortality % | 2,78 | 1,49 | - | 1,90 | 1,96 | - | 2,21 | 1,97 | - | 1,24 | 1,25 | - | -6,39 | -2,59 | - |
|  | Excess mortality rate | 2,26 | 1,78 | - | 1,38 | 1,44 | - | 1,26 | 1,26 | - | 1,09 | 1,11 | - | 0,72 | 0,87 | - |

**B**

|  |  | **< 65** |  |  | **65 - 69** |  |  | **70 - 74** |  |  | **75 - 79** |  |  | **> 80** |  |  |
| --- | --- | --- | --- | --- | --- | --- | --- | --- | --- | --- | --- | --- | --- | --- | --- | --- |
| **Women** |  | Period 1 | Period 2 | Period 3 | Period 1 | Period 2 | Period 3 | Period 1 | Period 2 | Period 3 | Period 1 | Period 2 | Period 3 | Period 1 | Period 2 | Period 3 |
| 1 year | N | 230 | 331 | 197 | 299 | 498 | 350 | 2102 | 3302 | 1740 | 332 | 659 | 431 | 187 | 451 | 386 |
|  | Observed mortality | 15 | 12 | 7 | 33 | 48 | 22 | 254 | 276 | 108 | 57 | 79 | 45 | 50 | 81 | 51 |
|  | Expected mortality | 1,4 | 1,9 | 1,1 | 3,7 | 5,2 | 3,5 | 85,5 | 106,9 | 49,5 | 10,4 | 19 | 11,3 | 11,9 | 27,4 | 24 |
|  | Excess mortality (O-E) | 13,6 | 10,1 | 5,9 | 29,3 | 42,8 | 18,5 | 168,5 | 169,1 | 58,5 | 46,6 | 60 | 33,7 | 38,1 | 53,6 | 27 |
|  | Excess mortality % | 5,91 | 3,05 | 2,99 | 9,80 | 8,59 | 5,29 | 8,02 | 5,12 | 3,36 | 14,04 | 9,10 | 7,82 | 20,37 | 11,88 | 6,99 |
|  | Excess mortality rate | 10,7 | 6,3 | 6,4 | 8,9 | 9,2 | 6,3 | 3,0 | 2,6 | 2,2 | 5,5 | 4,2 | 4,0 | 4,2 | 3,0 | 2,1 |
| 3 year | N | 209 | 310 | 143 | 255 | 440 | 235 | 1743 | 2863 | 1175 | 250 | 549 | 290 | 130 | 349 | 242 |
|  | Observed mortality | 3 | 7 | 4 | 8 | 23 | 11 | 107 | 158 | 50 | 19 | 39 | 18 | 15 | 23 | 21 |
|  | Expected mortality | 1,6 | 2 | 0,8 | 4,1 | 5,7 | 2,5 | 88,5 | 113 | 37,3 | 11,1 | 20,8 | 8,7 | 12,4 | 29,8 | 17,1 |
|  | Excess mortality (O-E) | 1,4 | 5 | 3,2 | 3,9 | 17,3 | 8,5 | 18,5 | 45 | 12,7 | 7,9 | 18,2 | 9,3 | 2,6 | -6,8 | 3,9 |
|  | Excess mortality % | 0,67 | 1,61 | 2,24 | 1,53 | 3,93 | 3,62 | 1,06 | 1,57 | 1,08 | 3,16 | 3,32 | 3,21 | 2,00 | -1,95 | 1,61 |
|  | Excess mortality rate | 1,88 | 3,50 | 5,00 | 1,95 | 4,04 | 4,40 | 1,21 | 1,40 | 1,34 | 1,71 | 1,88 | 2,07 | 1,21 | 0,77 | 1,23 |
| 5 year | N | 194 | 289 | 62 | 237 | 393 | 75 | 1512 | 2551 | 465 | 213 | 465 | 104 | 112 | 290 | 77 |
|  | Observed mortality | 7 | 17 | 0 | 18 | 20 | 1 | 97 | 198 | 26 | 19 | 48 | 11 | 15 | 36 | 8 |
|  | Expected mortality | 1,7 | 2,1 | 0,3 | 4,5 | 6,1 | 0,9 | 90,3 | 119,1 | 15,1 | 11,9 | 21,8 | 3,3 | 13,2 | 30,1 | 5,5 |
|  | Excess mortality (O-E) | 5,3 | 14,9 | -0,3 | 13,5 | 13,9 | 0,1 | 6,7 | 78,9 | 10,9 | 7,1 | 26,2 | 7,7 | 1,8 | 5,9 | 2,5 |
|  | Excess mortality % | 2,73 | 5,16 | -0,48 | 5,70 | 3,54 | 0,13 | 0,44 | 3,09 | 2,34 | 3,33 | 5,63 | 7,40 | 1,61 | 2,03 | 3,25 |
|  | Excess mortality rate | 4,12 | 8,10 | 0,00 | 4,00 | 3,28 | 1,11 | 1,07 | 1,66 | 1,72 | 1,60 | 2,20 | 3,33 | 1,14 | 1,20 | 1,45 |
| 10 year | N | 151 | 189 | - | 157 | 232 | - | 921 | 1393 | - | 130 | 205 | - | 47 | 93 | - |
|  | Observed mortality | 9 | 8 | - | 17 | 24 | - | 99 | 134 | - | 20 | 26 | - | 6 | 22 | - |
|  | Expected mortality | 1,8 | 2,1 | - | 4,5 | 5,6 | - | 78,6 | 106,6 | - | 11,9 | 16,8 | - | 8,7 | 13,8 | - |
|  | Excess mortality (O-E) | 7,2 | 5,9 | - | 12,5 | 18,4 | - | 20,4 | 27,4 | - | 8,1 | 9,2 | - | -2,7 | 8,2 | - |
|  | Excess mortality % | 4,77 | 3,12 | - | 7,96 | 7,93 | - | 2,21 | 1,97 | - | 6,23 | 4,49 | - | -5,74 | 8,82 | - |
|  | Excess mortality rate | 5,00 | 3,81 | - | 3,78 | 4,29 | - | 1,26 | 1,26 | - | 1,68 | 1,55 | - | 0,69 | 1,59 | - |

**Supplemental Table 3.** Cumulative incidence of cardiovascular death, with non-cardiovascular death as competing risk, per period. Stratified by age. A: men B: women.

**A**

|  | **Period 1**  (1995 – 2000) | **Period 2**  (2001 – 2011) | **Period 3**  (2012 – 2017) |
| --- | --- | --- | --- |
| **All** |  |  |  |
| 1 year | 6.09 (5.84 – 6.34) | 6.09 (5.84 – 6.34) | 6.09 (5.85 – 6.35) |
| 3 years | 9.49 (9.18 – 9.80) | 9.49 (9.18 – 9.80) | 9.50 (9.19 – 9.81) |
| 5 years | 13.02 (12.70 – 13.43) | 13.06 (12.69 – 13.43) | 13.10 (12.73 – 13.47) |
| 10 years | 22.14 (21.66 – 22.62) | 22.13 (21.65 – 22.62) | - |
|  |  |  |  |
| **< 65 years** |  |  |  |
| 1 year | 6.07 (5.82 – 6.32) | 6.13 (5.88 – 6.38) | 6.08 (5.84 – 6.34) |
| 3 years | 9.47 (9.16 – 9.78) | 9.41 (9.10 – 9.72) | 9.45 (9.14 – 9.76) |
| 5 years | 13.01 (12.65 – 13.38) | 13.11 (12.75 – 13.48) | - |
| 10 years | 22.14 (21.66 – 22.62) | 22.21 (21.73 – 22.70) | - |
|  |  |  |  |
| **65 - 69 years** |  |  |  |
| 1 year | 6.11 (5.86 – 6.36) | 6.08 (5.84 – 6.34) | 6.06 (5.81 – 6.31) |
| 3 years | 9.52 (9.22 – 9.84) | 9.49 (9.18 – 9.80) | 9.43 (9.12 – 9.74) |
| 5 years | 13.05 (12.69 – 13.42) | 13.05 (12.69 – 13.42) | - |
| 10 years | 22.17 (21.69 – 22.66) | 22.13 (21.65 – 22.62) | - |
|  |  |  |  |
| **70 - 74 years** |  |  |  |
| 1 year | 6.08 (5.83 – 6.33) | 6.13 (5.89 – 6.39) | 6.10 (5.85 – 6.35) |
| 3 years | 9.49 (9.18 – 9.80) | 9.48 (9.17 – 9.79) | 9.82 (9.51 – 10.14) |
| 5 years | 13.06 (12.70 – 13.43) | 13.06 (12.69 – 13.42) | - |
| 10 years | 22.14 (21.66 – 22.63) | 22.16 (21.68 – 22.64) | - |
|  |  |  |  |
| **75 – 79 years** |  |  |  |
| 1 year | 6.09 (5.84 – 6.34) | 6.08 (5.83 – 6.33) | 6.05 (5.81 – 6.30) |
| 3 years | 9.51 (9.20 – 9.82) | 9.49 (9.18 – 9.80) | 9.55 (9.24 – 9.86) |
| 5 years | 13.06 (12.70 – 13.43) | 13.08 (12.72 – 13.45) | - |
| 10 years | 22.11 (21.63 – 22.60) | 22.12 (21.64 – 22.61) | - |
|  |  |  |  |
| **> 80 years** |  |  |  |
| 1 year | 6.15 (5.91 – 6.41) | 6.09 (5.84 – 6.34) | 6.09 (5.85 – 6.35) |
| 3 years | 9.51 (9.20 – 9.82) | 9.50 (9.20 – 9.81) | 9.50 (9.19 – 9.81) |
| 5 years | 13.05 (12.69 – 13.42) | 13.10 (12.73 – 13.47) | - |
| 10 years | 22.12 (21.64 – 22.61) | 22.30 (21.82 – 22.79) | - |

**B**

|  | **Period 1**  (1995 – 2000) | **Period 2**  (2001 – 2011) | **Period 3**  (2012 – 2017) |
| --- | --- | --- | --- |
| **All** |  |  |  |
| 1 year | 8.24 (7.53 – 8.99) | 8.19 (7.48 – 8.93) | 8.17 (7.46 – 8.91) |
| 3 years | 12.49 (11.62 – 13.40) | 12.45 (11.58 – 13.35) | 12.47 (11.60 – 13.37) |
| 5 years | 17.14 (16.10 – 18.21) | 17.22 (16.18 – 18.28) | 16.86 (15.84 – 17.92) |
| 10 years | 28.01 (26.67 – 29.38) | 27.99 (26.64 – 29.34) | - |
|  |  |  |  |
| **< 65 years** |  |  |  |
| 1 year | 8.06 (7.36 – 8.80) | 8.84 (8.10 – 9.60) | 9.46 (8.70 – 10.25) |
| 3 years | 12.30 (11.43 – 13.20) | 12.66 (11.78 – 13.58) | 11.56 (10.72 – 12.43) |
| 5 years | 16.48 (15.46 – 17.52) | 16.21 (15.20 – 17.24) | - |
| 10 years | 28.02 (26.67 – 29.38) | 28.30 (26.95 – 29.67) | - |
|  |  |  |  |
| **65 - 69 years** |  |  |  |
| 1 year | 8.55 (7.83 – 9.31) | 8.20 (7.50 – 8.95) | 8.50 (7.78 – 9.26) |
| 3 years | 12.60 (11.72 – 13.51) | 12.34 (11.47 – 13.24) | 12.10 (11.25 – 13.00) |
| 5 years | 17.04 (16.01 – 18.10) | 16.89 (15.86 – 17.95) | - |
| 10 years | 28.71 (27.35 – 30.09) | 27.92 (26.58 – 29.28) | - |
|  |  |  |  |
| **70 - 74 years** |  |  |  |
| 1 year | 8.41 (7.69 – 9.16) | 8.26 (7.55 – 9.01) | 9.38 (8.63 – 10.17) |
| 3 years | 12.60 (11.72 – 13.51) | 12.45 (11.58 – 13.35) | 12.47 (11.60 – 13.37) |
| 5 years | 17.07 (16.03 – 18.13) | 17.24 (16.20 – 18.31) | - |
| 10 years | 28.14 (26.79 – 29.50) | 27.95 (26.61 – 29.31) | - |
|  |  |  |  |
| **75 – 79 years** |  |  |  |
| 1 year | 8.24 (7.53 – 8.99) | 8.22 (7.51 – 8.97) | 8.17 (7.46 – 8.91) |
| 3 years | 12.49 (11.62 – 13.40) | 12.64 (11.76 – 13.55) | 12.68 (11.80 – 13.60) |
| 5 years | 16.94 (15.91 – 18.00) | 17.22 (16.18 – 18.28) | - |
| 10 years | 28.36 (27.01 – 29.73) | 27.99 (26.64 – 29.34) | - |
|  |  |  |  |
| **> 80 years** |  |  |  |
| 1 year | 7.93 (7.23 – 8.66) | 8.19 (7.48 – 8.93) | 8.41 (7.69 – 9.16) |
| 3 years | 12.43 (11.55 – 13.33) | 12.53 (11.66 – 13.44) | 12.53 (11.66 – 13.44) |
| 5 years | 17.14 (16.10 – 18.21) | 17.27 (16.22 – 18.33) | - |
| 10 years | 28.39 (27.04 – 29.76) | 27.67 (26.33 – 29.02) | - |

**Supplemental Table 4.** Generalized mixed model. Effect of Charlson Comorbidity Index (CCI) on relative survival.

|  | **CCI 1** | **CCI 2** | **CCI > 3** |
| --- | --- | --- | --- |
| **Men** |  |  |  |
| <65 | 1.43 (1.17 – 1.76) | 2.75 (2.15 – 3.52) | 5.55 (4.34 – 7.10) |
| 65 – 70 | 1.52 (1.24 – 1.85) | 2.68 (2.13 – 3.38) | 6.07 (4.93 – 7.47) |
| 71 – 74 | 1.99 (1.53 – 2.59) | 3.55 (2.68 – 4.71) | 5.45 (4.16 – 7.16) |
| 75 – 79 | 2.82 (1.93 – 4.12) | 5.67 (3.84 – 8.37) | 11.81 (8.16 – 17.09) |
| > 80 | 5.61 (2.46 – 12.78) | 11.05 (4.79 – 25.48) | 23.39 (10.31 – 53.05) |
| **Women** |  |  |  |
| < 65 | 1.19 (0.82 – 1.74) | 1.93 (1.11 – 3.33) | 5.16 (3.08 – 8.64) |
| 65 – 70 | 1.16 (0.86 – 1.59) | 2.01 (1.39 – 2.90) | 4.92 (3.45 – 7.00) |
| 71 – 74 | 1.38 (1.01 – 1.89) | 2.52 (1.73 – 3.67) | 2.71 (1.79 – 4.10) |
| 75 – 79 | 1.67 (1.18 – 2.37) | 2.35 (1.53 – 3.61) | 5.08 (3.41 – 7.57) |
| > 80 | 2.73 (1.45 – 5.13) | 4.14 (2.06 – 8.32) | 8.63 (4.54 – 16.39) |

Odds ratio (OR) with 95% CIs. CCI 0 is reference.

**Supplemental Table 5.** Smoking habits of the general population in the Netherlands between 1995 – 2017.

| **Year** | **Proportion of smokers** |
| --- | --- |
| 1995 | 35.9 |
| 1996 | 36.0 |
| 1997 | 35.8 |
| 1998 | 34.7 |
| 1999 | 34.0 |
| 2000 | 32.7 |
| 2001 | 34.7 |
| 2002 | 33.7 |
| 2003 | 32.2 |
| 2004 | 31.1 |
| 2005 | 31.1 |
| 2006 | 31.1 |
| 2007 | 29.3 |
| 2008 | 29.0 |
| 2009 | 28.4 |
| 2010 | 26.8 |
| 2011 | 26.8 |
| 2012 | 24.4 |
| 2013 | 24.4 |
| 2014 | 25.4 |
| 2015 | 26.0 |

Statline data for the general Dutch population.1^4^ Data for the AAA population was not available.

**Supplemental Figure 1.** Cause of death distribution between 1995 – 2017 of AAA patients (A) versus the general population (B) by sex.


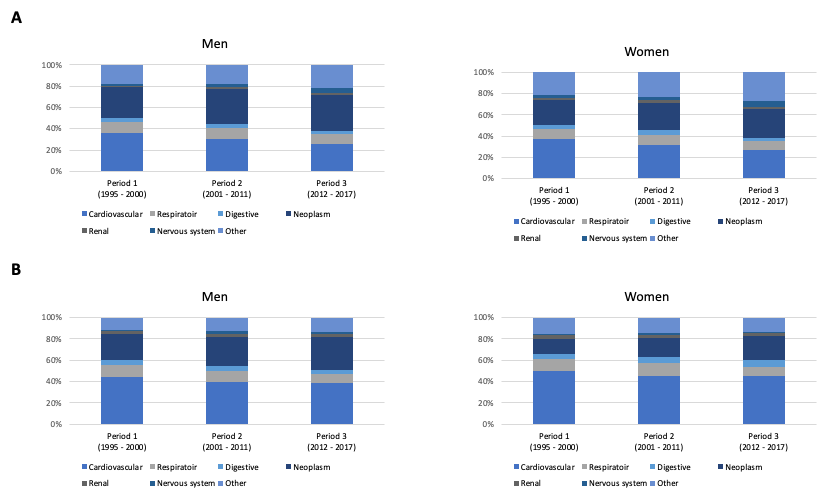


**Supplemental Figure 2.** Cumulative incidence of cardiovascular versus non-cardiovascular mortality over time (1995 – 2017) stratified by sex and age.


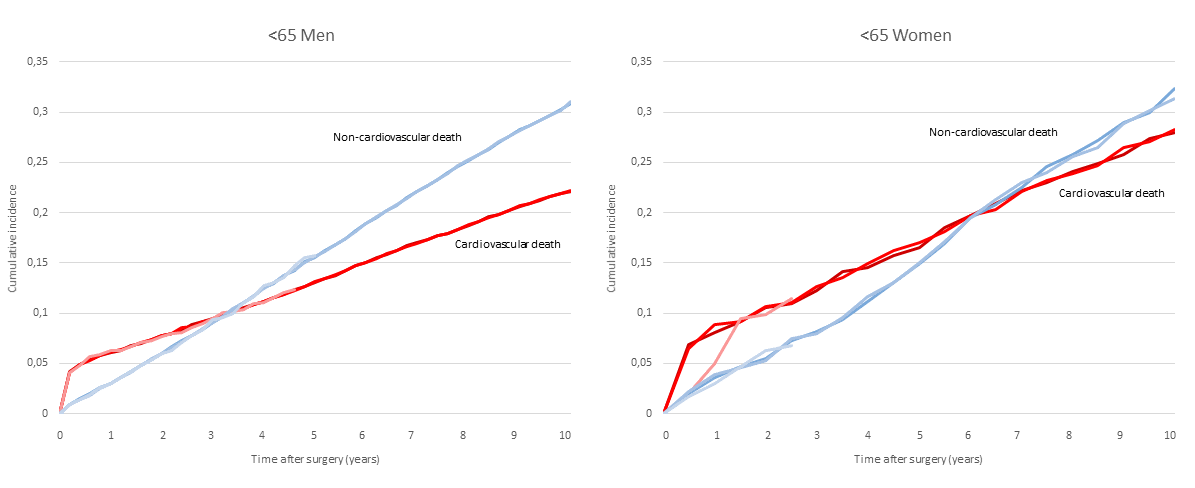


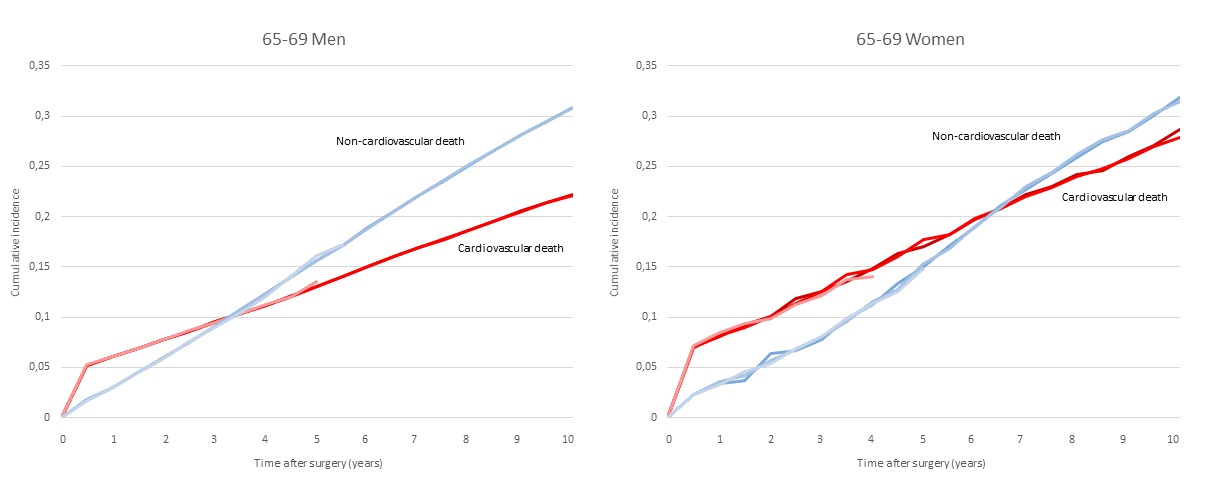


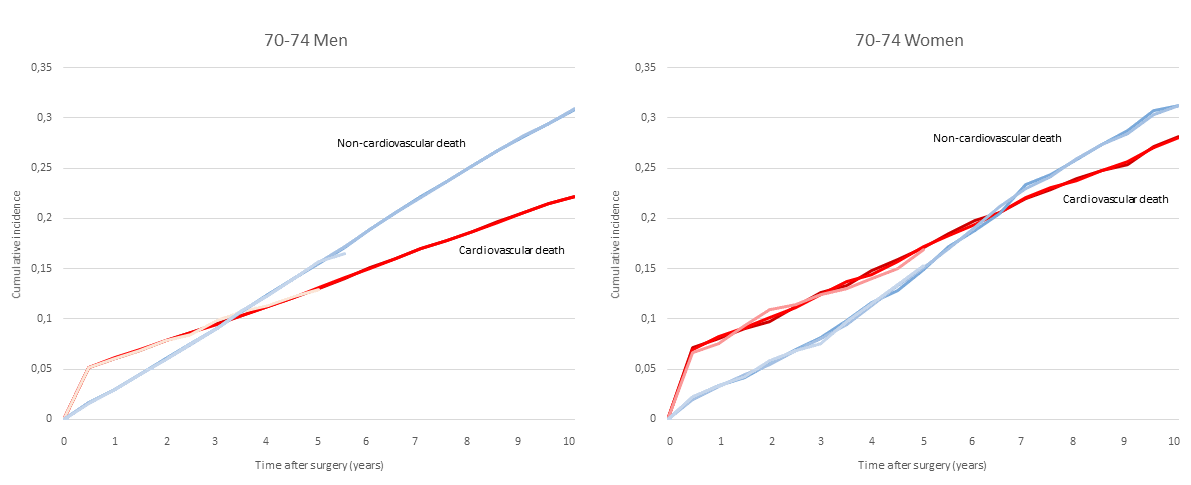


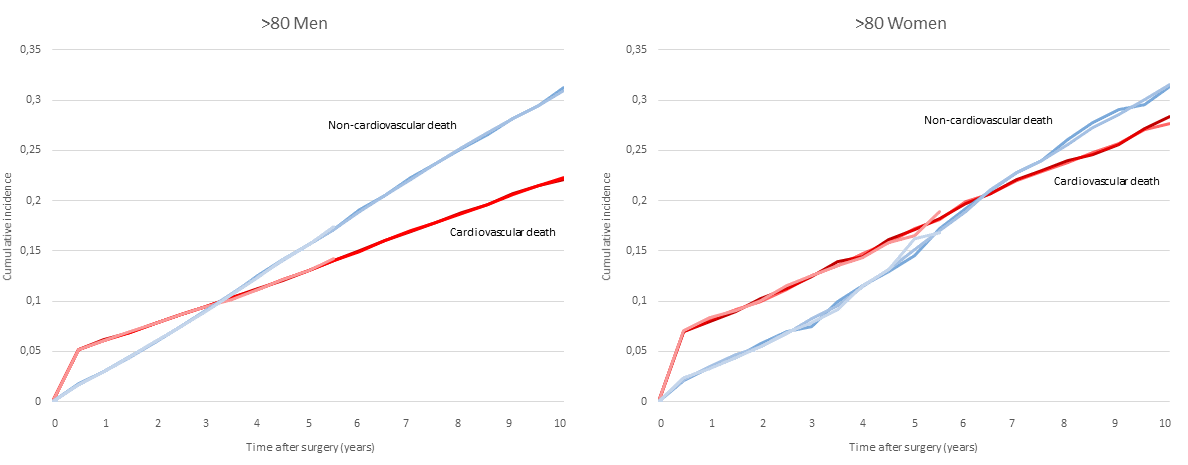

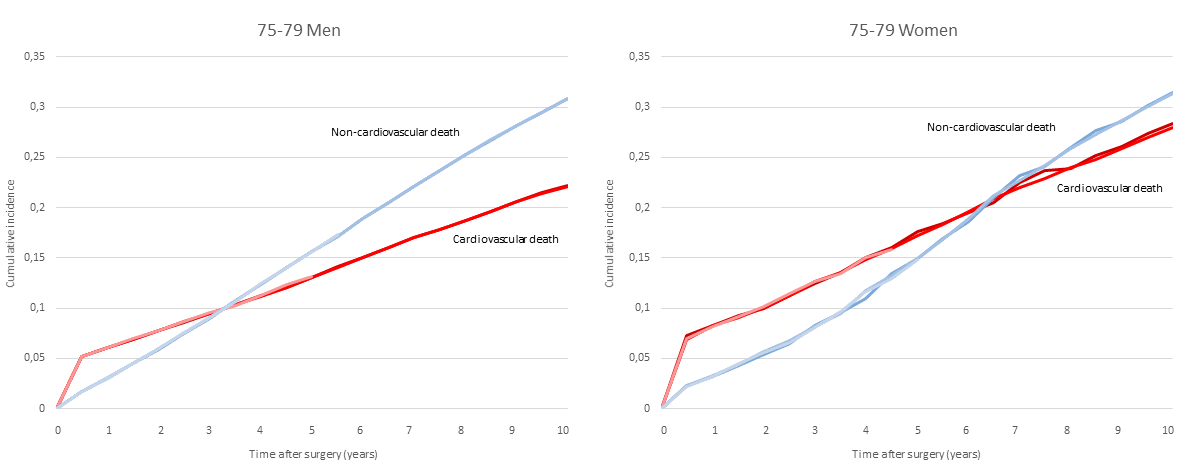


**Supplemental Figure 3.** Comparison between Dutch and Swedish population of Relative survival (RS) of patients after elective AAA repair between 2001 – 2015 by male and female at 1, 4, and 10 years.


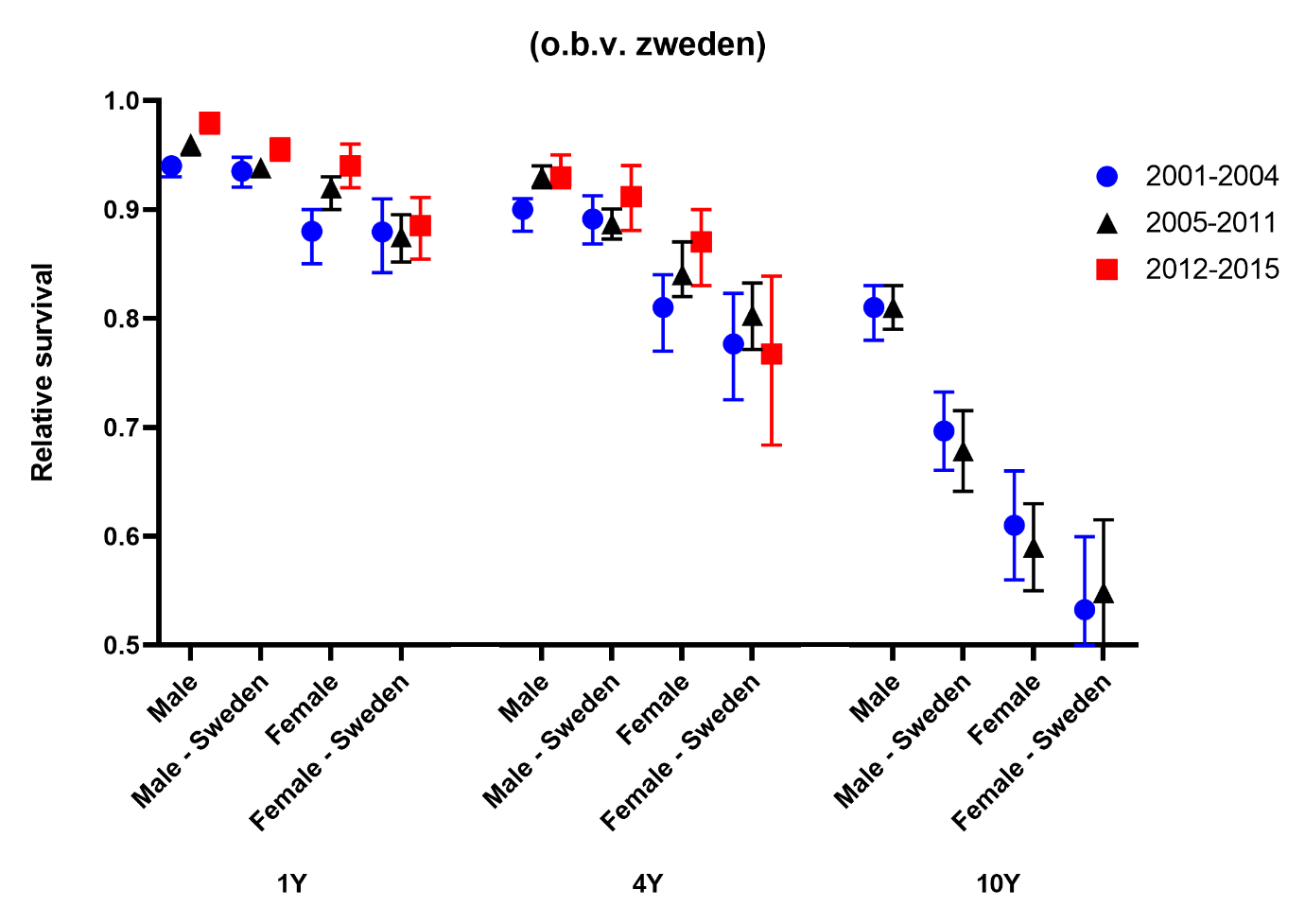


**REFERENCES**

1. Central Bureau of Statistics. Microdata: zelf onderzoek doen [in Dutch]. https://www.cbs.nl/nl-nl/onze-diensten/maatwerk-en-microdata/microdata-zelf-onderzoek-doen [↑](#endnote-ref-1)
2. Mackenbach JP, Van Duyne WM, Kelson MC. Certification and coding of two underlying causes of death in The Netherlands and other countries of the European Community. *J Epidemiol Community Health*. 1987;41:156-160. [↑](#endnote-ref-2)
3. von Elm E, Altman DG, Egger M, et. al. The Strengthening the Reporting of Observational Studies in Epidemiology (STROBE) statement: guidelines for reporting observational studies. *Lancet*. 2007;370:1453-1457. [↑](#endnote-ref-3)
4. Charlson ME, Pompei P, Ales KL, et. al. A new method of classifying prognostic comorbidity in longitudinal studies: development and validation. *J Chronic Dis*. 1987;40:373-383.  [↑](#endnote-ref-4)
5. Quan H, Li B, Couris CM, et. al. Updating and validating the Charlson comorbidity index and score for risk adjustment in hospital discharge abstracts using data from 6 countries. *Am J Epidemiol*. 2011;173:676-682.  [↑](#endnote-ref-5)
6. Radovanovic D, Seifert B, Urban P, et. al. Validity of Charlson Comorbidity Index in patients hospitalised with acute coronary syndrome. Insights from the nationwide AMIS Plus registry 2002-2012. *Heart*. 2014;100:288-294.  [↑](#endnote-ref-6)
7. Prinssen M, Buskens E, Blankensteijn J. The Dutch Randomised Endovascular Aneurysm Management (DREAM) trial. Background, design and methods. The Journal of cardiovascular surgery. 2002;43:379-84. [↑](#endnote-ref-7)
8. Gezondheidsraad. Bevolkingsonderzoek naar aneurysma van de abdominal aorta (AAA) [in Dutch]. 2019. Available at: <file:///Users/ruthbulder/Downloads/kernadvies+Bevolkingsonderzoek+naar+aneurysma+van+de+abdominale+aorta+AAA.pdf> (data downloaded on [15-03-2021]) [↑](#endnote-ref-8)
9. Dutch Surgical Aneurysm Audit database. Data available for 2013 – 2019. [↑](#endnote-ref-9)
10. Bastiaannet E, Liefers GJ, de Craen AJ, et. al. Breast cancer in elderly compared to younger patients in the Netherlands: stage at diagnosis, treatment and survival in 127,805 unselected patients. Breast *Cancer Res Treat*. 2010;124:801-807. [↑](#endnote-ref-10)
11. Rutherford MJ, Dickman PW, Lambert PC. Comparison of methods for calculating relative survival in population-based studies. *Cancer Epidemiol*. 2012;36:16-21. [↑](#endnote-ref-11)
12. de Glas NA, Kiderlen M, Vandenbroucke JP, et. al. Performing Survival Analyses in the Presence of Competing Risks: A Clinical Example in Older Breast Cancer Patients. *J Natl Cancer Inst*. 2015;108:djv366. [↑](#endnote-ref-12)
13. Human Mortality Database. University of California BU, and Max Planck Institute for Demographic Research (Germany). Available at [www.mortality.org](file:///C:\Users\jc138185\AppData\Local\Microsoft\Windows\INetCache\Content.Outlook\MRUPLDC3\www.mortality.org) or [www.humanmortality.de](file:///C:\Users\jc138185\AppData\Local\Microsoft\Windows\INetCache\Content.Outlook\MRUPLDC3\www.humanmortality.de) (data downloaded on [06-09-2022]).

    ^14^ Statline. Gezondheid, leefstijl, zorggebruik en -aanbod, doodsoorzaken; vanaf 1900 [in Dutch]. Available at <https://opendata.cbs.nl/#/CBS/nl/dataset/37852/table?searchKeywords=stoppen%20met%20roken> (data downloaded on [24-04-2023]). [↑](#endnote-ref-13)
